# A Review of the Integrated Model of Care: An Opportunity to Respond to Extensive Palliative Care Needs in Pediatric Intensive Care Units in Under-Resourced Settings

**DOI:** 10.3389/fped.2018.00003

**Published:** 2018-01-23

**Authors:** Michelle Grunauer, Caley Mikesell

**Affiliations:** ^1^Escuela de Medicina, Colegio de Ciencias de la Salud, Universidad San Francisco de Quito, Quito, Ecuador; ^2^Pediatric Intensive Care Unit, Hospital de los Valles, Quito, Ecuador

**Keywords:** pediatric palliative care, integrated model of care, pediatric critical care, pediatric intensive care, Pediatric Palliative Screening Scale, low-resource settings, consultative model

## Abstract

It is estimated that 6.3 million children who die annually need pediatric palliative care (PPC) and that only about 10% of them receive the attention they need because about 98% of them live in under-resourced settings where PPC is not accessible. The consultative model and the integrated model of care (IMOC) are the most common strategies used to make PPC available to critically ill children. In the consultative model, the pediatric intensive care unit (PICU) team, the patient, or their family must request a palliative care (PC) consultation with the external PC team for a PICU patient to be evaluated for special care needs. While the consultation model has historically been more popular, issues related to specialist availability, referral timing, staff’s personal biases, misconceptions about PC, and other factors may impede excellent candidates from receiving the attention they need in a timely manner. Contrastingly, in the IMOC, family-centered care, PC tasks, and/or PC are a standard part of the treatment automatically available to all patients. In the IMOC, the PICU team is trained to complete critical and PC tasks as a part of normal daily operations. This review investigates the claim that the IMOC is the best model to meet extensive PPC needs in PICUs, especially in low-resource settings; based on an extensive review of the literature, we have identified five reasons why this model may be superior. The IMOC appears to: (1) improve the delivery of PPC and pediatric critical care, (2) allow clinicians to better respond to the care needs of patients and the epidemiological realities of their settings in ways that are consistent with evidence-based recommendations, (3) facilitate the universal delivery of care to all patients with special care needs, (4) maximize available resources, and (5) build local capacity; each of these areas should be further researched to develop a model of care that enables clinicians to provide pediatric patients with the highest attainable standard of health care. The IMOC lays out a pathway to provide the world’s sickest, most vulnerable children with access to PPC, a human right to which they are entitled by international legal conventions.

## Palliative Care (PC) in the Intensive Care Unit (ICU)

The silo thinking that underpins much of western medicine cannot universally facilitate the enforcement of the human right to the highest possible standard of health care and, in many cases, may be antagonistic to the pursuit of that goal; ICUs in under-resourced settings characterized by great health-care-related inequalities, poor infrastructure, and limited personnel are one such context in which silo thinking cannot structure care to appropriately meet the multifaceted needs of patients (1). The tendency to isolate medical specialties under this framework has led to a division between intensive and PC—two branches of medicine that are essential to the treatment of critically ill patients in ICUs. This common practice has contributed to the perception that adding PC tasks to intensivists’ daily responsibilities will burden them, unnecessarily absorb valuable time and resources, and take away from their “real care goals.” On the contrary, a breadth of evidence shows that adopting a holistic care strategy can conserve valuable resources in the ICU (2–4) and improve patients’, their families’, and caretakers’ well-being (5).

Silo thinking is the foundation of the most popular strategy used to make PC available in the ICU: the consultative model (5). In purely consultative models, the hospital uses its resources to hire additional specialists and perhaps even create a stand-alone PC department. This external team is brought into the ICU to care for patients who are identified as having PC needs. The patient, their family, or their health-care providers must first identify the need for PC and then request a consultation with the external PC team for such attention to be provided.

According to the consultative model, ICU staff are positioned as gatekeepers to PC access; if they lack training, have negative attitudes or false beliefs about PC, do not understand its applications and potential benefits, or cannot recognize the “triggers” that signal a patient’s PC needs, external PC resources may not be used when indicated and, consequently, PC may not be normalized in a given ICU (6, 7). Lynn explains, “The course of care is much more strongly associated with the service supply and habit patterns of the local care system than with the particular preferences or prognoses of the individual patient” (8). If consultative models do not involve the intentional training of ICU staff in holistic care, the personnel’s attitudes, knowledge, and practices may impede the treatment of patients’ PC needs (6, 7).

Contrastingly, in the integrated model of care (IMOC), family-centered care, PC tasks, and/or PC are a standard part of the treatment automatically available to all patients. In the IMOC, the ICU team is trained in holistic critical care and PC tasks are embedded in the unit’s protocols. As a part of normal daily operations, team members simultaneously initiate PC and critical care, screen for PC needs regularly, and design plans to meet those needs as they arise. Although the consultative model is overall more common (5), the IMOC appears to offer distinct benefits to patients with pediatric palliative care (PPC) needs and, in pediatric ICUs, “is rapidly becoming the standard for high quality care of critically ill children” (4). While both models can make PC available in ICUs and pediatric intensive care units (PICUs), the IMOC may be more beneficial to patients/their families and may be better suited to under-resourced settings where hiring outside specialists may be impossible.

To date, there is little research that directly compares the effectiveness of the IMOC and the consultative model. However, one compelling 2011 study comparing models of PC in the United States found family members’ opinions of their loved ones’ last month of life correlated with the model according to which they were treated. The family of patients who had received integrated PC services were the most likely to report that the deceased had an “excellent” quality of life during their last month, followed by those who had received consultative PC services, and then by those who had only received usual care (no PC) (9). The group that received integrated PC services also appeared to receive more holistic attention; they were more likely to have accessed chaplain services and prepared do not resuscitate orders before death; their families also received more bereavement contact following their passing (9). This study presents direct evidence that the IMOC may be superior to the consultative model across various dimensions of care (9), even in industrialized countries where more PC specialists, superior infrastructure, greater education, and more medical resources are available (10).

This review investigates the claim that the IMOC is the best model to meet extensive PPC needs in PICUs, especially in low-resource settings; based on an extensive review of the literature, we have identified five reasons why this model may be superior. The IMOC appears to: (1) improve the delivery of PPC and critical care, (2) allow clinicians to better respond to the care needs of patients and the epidemiological realities of their settings in ways that are consistent with evidence-based recommendations, (3) facilitate the universal delivery of care to all patients with special care needs, (4) maximize available resources, and (5) build local capacity in a way that responds to the current shortage of PC specialty education; each of these areas requires further investigation to improve PPC delivery.

This review integrates evidence from countries around the world, but especially focuses on under-resourced settings like Ecuador, the authors’ country of residence. The need for improved PPC and pediatric critical care (PCC) delivery is particularly urgent in contexts like ours because the high burden of PPC needs overwhelms our underdeveloped health-care infrastructure. Unfortunately, as this review will highlight, the majority of PC-related studies have been completed in industrialized countries and, as such, do not always accurately reflect the realities of PC and health-care professionals in our context. The skewed perspective of the academic discipline of PC was evidenced by a 2016 review of the last 20 years of publications in the field of PC, which revealed that over 90% of all documents published on the topic originated in industrialized nations classified as Group 4 countries by the World Palliative Care Alliance, meaning that they have already achieved integration of hospice-PC into their health-care systems (10, 11). When industrialized countries’ practices, beliefs, and customs are assumed to be the neutral standard for medical care and subsequently applied to different cultural contexts, health professionals may disregard the unique assets, traditions, and needs of under-resourced communities in which they work. Such dynamics have the potential to further oppress already disadvantaged groups, waste resources, and create additional barriers between PC and the patients who need it. For this reason, we integrate as much pertinent data from our context as possible, but significantly supplement it with findings from industrialized nations.

## Resistance to PC in the ICU

The direct integration of PC into the PICU/ICU through the IMOC is commonly met with resistance because of prevalent myths, discipline-based bias, and a lack of knowledge of evidence-based recommendations (12, 13). Although the specific barriers to implementing PC in the ICU vary from culture to culture and unit to unit, some of the particularly problematic misconceptions that keep this specialty out of the ICU are the ideas that PC is (1) ineffective and unimportant for most ICU patients, (2) synonymous with hospice and hopelessness on the part of the family, patients, and/or clinician, (3) equivalent to the “soft skills” that health-care professionals already innately have, and (4) wasteful in that it absorbs valuable time and resources from intensivists. Although prevalent, none of these ideas are consistent with decades of research about PC. Table 1 provides a summary of key articles highlighting the importance of the PC in the ICU.

**Table 1:** Summary of key articles.

Reference	Summary
Nelson et al. (14)	Rapid response teams (RRTs) or emergency medical teams are common in intensive care unit (ICU) settings and seek to prevent morbidity and mortality among already hospitalized patients who may be deteriorating. While there are conflicting data about the effectiveness of RRTs in accomplishing these goals, RRTs are well positioned to meet the palliative care (PC) needs of patients in the ICU. RRTs can, and in many cases, already perform a range of PC tasks related to communication, emotional/psychological support, symptom control, and pain management. RRTs should also be given the tools necessary to facilitate family conferences in emergencies, provide family-centered care, support distressed caregivers, foster shared decision-making, and help colleagues to administer measures of self-care. Considering their already extensive skillsets and proximity to patients, their families, and professional caretakers, RTTs should be trained to provide PC to patients admitted to ICUs and emergency departments
Boss et al. (4)	This article reviews the benefits that patients, their families, and pediatric intensive care unit (PICU) staff experience “when PC is intentionally incorporated into the PICU.” (4) PC is indicated in a large share of children admitted to PICUs; as epidemiological shifts occur across the world, an increasingly large number of children become eligible for PC to address the needs that arise from their complex, chronic diseases. PC is interdisciplinary and addresses a range of needs of patients, families, and their professional caregivers, including pain management, symptom control, family and patient-centered communication and decision-making, psychological care, and other types of support key to the well-being of patients, their families, and PICU personnel. This review also highlights some of the gaps in care in PICUs, including accurate pain assessment, poor communication between families and PICU staff, inadequate psychosocial treatment, high levels of unaddressed psychological morbidity among family members, and other issues; intentionally integrating PC into PICUs is an excellent opportunity to meet such needs
O’Brien et al. (15)	This article examines the potential impact and methodology for a future intervention in 26 PICUs from Canada, Australia, and New Zealand to change the role of parents in neonatal care. Based on data from previous similar interventions, O’Brien et al. hypothesized that integrating parents into the NICU team as providers of all, but the most advanced medical interventions would result in faster weight gain, greater rates of breastfeeding, and improved clinical outcomes for infants as well as reduced levels of stress and anxiety for parents. The FICare intervention program requires primary care givers to undergo extensive training to learn how to properly care for their neonates, commit to 6 h daily to caring for their babies, record their interactions with their babies in a special journal, and interact with “veteran parents,” who give personal support to parents whose babies are in the NICU. Previous data evidence the great positive potential for this intervention
Curtis (16)	ICU personnel should be trained to provide PC in their units because it plays an important role in the care of critically ill patients, not merely those at risk for dying. PC is important because it allows us to better facilitate intentional discussions about treatment plans, other types of communication, patient-focused/family-centered decision-making, symptom management, multidisciplinary collaborations in patient treatment, end-of-life decisions, logistical planning, and other aspects of care. Implementing PC in the ICU could help to address diverse unmet symptoms or patients and their families, improper communication techniques employed by ICU personnel, conflicts/lack of communication within the ICU, and many other difficulties. To improve PC in ICUs, Curtis suggests educating ICU personnel in PC and how to overcome PC implementation barriers, establishing institutional policies to promote PC, and providing ICU staff with feedback from families of their patients. PC should be implemented in the ICU to improve the experiences and well-being of patients, their families, and the ICU staff itself
Aslakson et al. (17)	PC is used to address the complex care needs of critically ill individuals, regardless of their prognoses or diagnoses; as such, this type of care should be initiated for various critically ill individuals upon ICU admission to better address psychosocial, spiritual, and physical symptom management; coordinate, plan and communicate about multidisciplinary treatment that reflects the patient’s and their family’s preferences; provide family-centered care and extensive care planning; and facilitate the family caregivers’ and ICU personnel’s own self-care. Aslakson et al. identified clinician’s subpar communication skills, unrealistic expectations related to patients and treatment, clinicians’ time constraints, decision-making difficulties, and other areas as opportunities for care improvement within the ICU which PC could address. The authors suggest that further research needs to be completed to determine the best methods for providing patients and their families with PC in situations of critical illness both inside and outside of the ICU

Contrary to the first myth, evidence shows that PC has major implications for the well-being of patients, their families, and practitioners (4, 5, 9, 18–25). PC is an interdisciplinary field that seeks to prevent and relieve the multifaceted suffering that critically ill patients and their families experience as a result of acute, chronic, life-limiting, and life-threatening conditions. PC addresses physical, psychosocial, ethical, spiritual, cultural, familial, and communication-related distress, as well as death and bereavement issues (21, 26–28). The timely initiation of PC is associated with (1) improved symptom management (18–20), (2) positive outcomes in patients (4, 18, 20), (3) improved quality of life for patients, families, and caregivers (18, 20), (4) better communication between professionals, patients, and their families (1, 4, 29, 30), and (5) longer life survival times among patients (4, 29).

Furthermore, PC belongs in the PICU/ICU because virtually all critically ill patients exhibit some level of PC need throughout their hospitalization (4, 5, 14, 31); to meet patients’ extensive PPC needs, PICUs may benefit from making PPC universally accessible through an IMOC. PC is indicated in a wide range of patients, not only those confronting the terminal stages of illness (4). Because PC is inaccurately equated with hospice, it is commonly and erroneously thought to signal imminent death and therefore may provoke anxiety among patients, families, and practitioners. Combined with the lack of knowledge about PC recommendations, this reputation fodders the incorrect idea that PC—care supposedly directed only at the dying—is not applicable to most patients in the ICU. However, the World Health Organization (WHO) (22), United Nations (32), and together for Short Lives’ PC charity (28) recommend that PPC should be extended to children with life-limiting conditions and not solely to those who are terminally ill. Cook and Rocker explain, “The coexistence of palliative care and critical care may seem paradoxical in the technological ICU. However, contemporary critical care should be as concerned with palliation as with the prevention, diagnosis, monitoring, and treatment of life-threatening conditions” (33). PC not only improves the quality of patients’ deaths but also of their lives.

Palliative care can be beneficial to a wide range of patients regardless their prognoses and diagnoses (16, 17, 22, 28). Boss et al. explain that PC should begin at the time of diagnosis of a potentially life-limiting condition and continue throughout the disease trajectory, regardless of the expected outcome (4). Although this type of care is particularly important to the terminally ill, it also plays a complementary role in the care of critically ill patients who may be able to make a complete recovery (22, 32). Together for Short Lives, a leading charity in the United Kingdom for children with life-limiting and life-threatening conditions, developed a categorization of pediatric conditions for which PC is indicated; these include potentially curable diseases (in which life is at risk and treatment may or may not succeed), progressive conditions, irreversible conditions causing severe disability, and morbidities that inevitably cause premature death (28). PC is applicable in all stages of such diseases and conditions (10, 34–39) and represents the evolution of hope, rather than the extinguishment of it—in some illness. Through impeccable assessment, treatment, and communication carried out by multidisciplinary teams, PC enhances the quality of life for a diverse range of patients and their loved ones, independent of what the future may hold for them (4, 5, 21, 22). Through PC, we can focus our hope on a better quality of life, death, and other realistic goals rather than wish for impossible outcomes.

In addition, considering that PC involves a range of multidisciplinary tasks to address various needs common among most PICU patients (4, 5, 31), health-care professionals should not be expected to be innately prepared to address all PC needs. Special training is required to address any one of the many areas that PC seeks to address in a way that is consistent with evidence-based recommendations. Unsurprisingly, several studies have shown that ICU staff’s beliefs about PC, knowledge of the field, and their actual practices in the ICU are deeply interconnected. One study of ICU nurses’ attitudes about PC found that participants had “moderately negative to neutral attitudes toward PC,” especially with regards to patient preferences and withholding/withdrawing treatment (7). Unsurprisingly, Razban et al. found that nurses’ beliefs were significantly correlated with their own “…personal study about palliative care, level of education, and experience of caring for a dying family member” (7). These findings suggest that education is key to creating an environment receptive to PC. Another study of nurses, intensivists, and advanced practice providers at three large academic hospitals found the “triggers” for initiating PC consultation to be an important barrier: the staff most frequently relied on triggers that resulted in late-stage PC application (6), which is inconsistent with best-practice recommendations that PC should be applied early and concurrently with disease-directed treatment (2, 22, 28). Again, education is key to implementing evidence-based practices; if ICU staff are expected to bridge the gaps in PC in their units, they must be properly trained to do so.

Finally, contrary to the popular myth that it is wasteful and unnecessarily burdensome for intensivists, PC is actually a resource-saving strategy in the ICU (2, 3). In under-resourced contexts like ours, distributive justice in medical care is perhaps the most prominent ethical principle that we encounter on a day-to-day basis. PC saves valuable resources because it prevents morbidities like depression and anxiety (3) and prevents futile treatments (2), thereby conserving resources to treat other patients. Well-managed PC patients are able to enjoy an improved quality of life, may have better outcomes, and are empowered to make long-term care plans if necessary; this, in turn, helps PC to maximize local resources on the population level because it prevents unnecessary emergency room visits and keeps precious ICU beds open (3). In countries with limited medication access, health-care personnel, and ICU capacity, PC stands to have a dramatic impact on the well-being of individuals and communities alike.

## PPC Across the Global South

In light of their effectiveness, the international community has declared PC and PPC basic human rights, maintaining that they are fundamental to the appropriate treatment of adults and children with special care needs in any stage of disease (20, 21, 23, 32, 35, 36, 39–42). Furthermore, “Palliative care is a recognized component of the right to the highest attainable standard of health, which is protected in article 12 of the International Covenant on Economic, Social and Cultural Rights, and in article 24 of the Convention on the Rights of the Child” (32). The WHO’s inclusive definition of PPC echoes this sentiment (22):

- PC for children is the active total care of the child’s body, mind, and spirit, and also involves giving support to the family.
- It begins when illness is diagnosed and continues regardless of whether or not a child receives treatment directed at the disease.
- Health providers must evaluate and alleviate a child’s physical, psychological, and social distress.
- Effective PC requires a broad multidisciplinary approach that includes the family and makes use of available community resources; it can be successfully implemented even if resources are limited.
- It can be provided in tertiary care facilities, in community health centers and even in children’s homes.

As described by the United Nations (43) and the WHO (22), the holistic focus of PPC makes it indispensable to achieving the highest attainable standard of care in many critical pediatric cases.

Despite the overwhelming evidence of its effectiveness, the vast majority of children who require PPC never receive it (10, 44); in part, this is because nearly 98% of pediatric patients who have PC needs come from low-middle income countries (LMICs) where it is simply not available (10). While many of these patients receive treatment directed at the disease, few receive concomitant PC (24, 25). Overall, around 6.3 million children requiring PPC die annually, but only about 10% of them receive the attention they need (10). The lack of PPC availability across the world results in the unnecessary suffering of the world’s most vulnerable children.

## PPC Need in Ecuador

To better elucidate the urgent need for improved, more accessible PPC in contexts like Ecuador, it is vital to identify aspects of our context which may result in more extensive PC needs (10) and which may be unexpected for readers from industrialized contexts. The combination of issues unique to LMICs may contribute to an overall higher need for PPC which might not be seen in industrialized countries, where PICUs may commonly receive many children who need an intermediate level of care or postoperative stabilization (4). The level of development and fragmentation of the Ecuadorian health-care system leads to the deterioration of patients before they arrive to the hospital and therefore contribute to a greater need for PC. These challenges can be grouped into three categories, including: (1) insufficient Infrastructure, (2) lack of trained specialists, and (3) socioeconomic factors (20, 44).

With respect to infrastructure issues, the number of PICU beds and emergency transport vehicles are major hurdles for providing appropriate, timely care. Ecuador only has 36 PICU beds available throughout the entire country (45); therefore, the patients who take priority for these few precious spots are often in extremely critical states. Because of the severity of these cases, the average national PICU mortality rate in 2012 was around 15% (as compared with a 5% mortality rate in European PICUs) (45). Similarly, Ecuador has a fragmented emergency transport system which contributes to the severity of cases that arrive to the PICU. Aside from a menagerie of ambulances of varying quality from the private sector, Ecuador only has 714 medical emergency transport vehicles from the public sector, which are insufficient to meet the population’s needs (46).

Similarly, a lack of trained pediatric intensivists and PC specialists in Ecuador makes it more difficult for children to receive the care they need. In Ecuador, there are approximately 33 pediatric intensivists (45) who are responsible for a pediatric population of nearly 5,000,000 (47). Unsurprisingly, the majority of these specialists are located in cities, further exacerbating regional inequalities in care.

Finally, socioeconomic factors reduce the quality of care available to pediatric patients. The lack of development of quality health care in rural areas, historical discrimination, poverty, and a lack of general health education especially among vulnerable groups are barriers (44). In addition, Ecuadorian children are also at great risk for comorbidities that can aggravate their conditions including parasitic infestations (48), malnutrition (49), and anemia (49). Similarly, the main causes of death common among Ecuadorian children include acute respiratory infections, traffic accidents, congenital malformations, leukemia, and other external causes, each of which can entail PPC needs before death (50, 51).

## The IMOC: A Pathway to Meet International PPC Needs

Considering the extensive PPC needs of PICU patients (especially in LMIC countries like Ecuador), the IMOC may be the best strategy to make PC available to all of those patients who may require it because the IMOC appears to (1) improve the delivery of PPC and PCC, (2) allow clinicians to better respond to the care needs of patients and the epidemiological realities of their settings in ways that are consistent with evidence-based recommendations, (3) facilitate the universal delivery of care to all patients with special care needs, (4) maximize available resources, and (5) build local capacity. The IMOC lays out a pathway to provide the world’s sickest, most vulnerable children with access to PPC, a human right to which they are entitled by international legal conventions (20, 22, 32, 34–39, 41).

The IMOC may improve the delivery of PPC in under-resourced environments because it bypasses those barriers which often impede the timely application of PPC. The advantage of the IMOC as compared with other models is that it already has PC embedded in its protocols. While the consultation model can make PPC available to patients, issues such as the timing of referral, physicians’ personal perspectives, ethical dilemmas, decision-making difficulties, lack of screening tools and trained personnel, communication issues, and misconceptions about PC can impede excellent candidates from receiving the attention they need in a timely manner (4, 52).

The IMOC also improves the delivery of PPC in settings like ours because it does not assume that patients, families, or even health-care workers innately know what PPC is and when to request a consultation for such care. In many countries, most families do not know of the existence of PPC or understand what it involves; thus, they may never ask for the care or even accept it when it is offered (53). Worse yet, some families and many medical professionals may believe damaging myths about PC, such as the erroneous idea that PC hastens death (12); a plethora of evidence, including the low mortality rate in our own center which uses the IMOC (4.8%) (54), discredits this notion. In an IMOC, the aforementioned obstacles are non-existent because all patients, regardless of their disease state, prognosis, and diagnosis, are regularly screened for PPC needs.

Moreover, we speculate that the IMOC is the best model to meet patients’ needs in the PICU because it better responds to epidemiological trends around the world and to patients’ needs in ways that are consistent with evidence-based recommendations. As epidemiological transitions occur across the world, an increasingly large share of patients with complex, chronic diseases are being seen more frequently and repeatedly in ICUs/PICUs; Lynn argues that current health systems that bifurcate curative treatment and PC do not adequately respond to patients’, families, and communities’ care needs in an appropriate or sustainable way (8). Rather, models of care ought to focus on early intervention, continuity, family-centeredness, and patients’ psychosocial suffering to create care pathways that help patients to avoid unnecessary suffering and that allow communities to conserve their precious medical resources (8, 15).

In addition, embedding PPC directly into PICU care models sidesteps the resistance against PC initiatives common in low-resource settings and maximizes local resources. Because the IMOC necessitates that PICU teams themselves are trained to carry out PC tasks, this model does not require additional staff or a new department as the consultation model does. PC consultation models are commonly met with resistance because cost-related hurdles can impede the establishment of new departments, pain and suffering are often normalized in low-resource settings (20), and PC may be viewed as a “luxury” for industrialized countries (20). By hybridizing PCC and PPC, it is possible to avoid such hurdles that can prevent the establishment of important PC programs.

Furthermore, the consultative PC model is ineffective in many settings because it relies on communities’ current capacity; the IMOC is more effective in meeting PC needs because it involves and trains all ICU personnel and therefore necessitates capacity-building in every unit in which it is implemented. Evidence indicates that the consultative model is limited in its capacity to meet communities’ PC needs even in resource-rich industrialized countries. For example, in a 2017 study, Wysham et al. reported that up to 35% of ICU patients in the United States needed PC; however, even in such a resource-rich setting, this level of need still overwhelmed the available 5,500 PC specialists’ capacity (6). In other words, even in the United States, there are not enough PC specialists to make the consultative model effective in addressing national PC need. Wysham et al. explain, “In this context, it is estimated that <5% of ICU patients receive specialist palliative care” (6).

The apparent dominance of the consultative PC model in ICUs seems even less appropriate when examined through the lens of pediatric intensive care in under-resourced settings like ours. Ecuador is categorized as a 3A country in the provision of PC (meaning that it has isolated provision in level of PC development) (10) and a high burden of conditions, diseases, and accidents that require PC (47, 49, 50). The burden of PC need combined with a lack of specialists renders the consultative model ineffective in addressing national and international PC needs.

While it is easy to point to a need for more training programs as a solution to the gross mismatch between PPC needs and specialist availability across the world, this idealistic thought process follows the paradigm that heralds the consultative model as king. It is true that there is great global need for more PPC training programs; however, international conventions entitle the world’s sickest children to pathways of care that provide more immediate access to the highest available standard of health care (which must include access to PC services) (22, 43). The IMOC proposes a paradigm shift that would make it possible to begin meeting children’s PC needs more rapidly and effectively than the consultative model.

Although trained PPC specialists may have greater expertise than health-care professionals who do not have the same level of formal training, many PICU personnel already unknowingly engage in some dimensions PC and can learn tasks to improve the level of care that they already provide (14). Shifting from the consultative model to the IMOC requires us to honestly appraise and develop the PC skills of all PICU/ICU staff, not only those of highly trained specialists. While a breadth of evidence demonstrates that beliefs and practices incongruent with the goals of PC abound in ICUs (6, 7, 34, 53), this does not indicate that intensive care staff lack the valuable foundations necessary to learn how better perform PC tasks. In Ecuador (45), the United States (6), and probably in most other settings, pediatric intensivists, nurses, and other support personnel present in PICUs greatly outnumber the trained PPC specialists. Whereas the consultative model frames this reality as damning, the IMOC frames it as a latent possibility for PC expansion. Ecuador has 33 pediatric intensivists (45) and countless other health-care professionals that work in PICUs—according to the IMOC, these health-care professionals ought to be considered potential PPC providers.

## The IMOC in Ecuador

While it may seem foolishly idealistic to paint non-specialist PICU/ICU personnel as potential PPC providers, in recent years our team has pioneered a number of training programs that hybridize PCC and PPC. In 4 years, these programs have disseminated the IMOC and increased the national Pediatric Critical Care and Emergency Medicine (PCCEM) and PPC capacity by 560% (54). Without making formal policy changes, gaining new specialists, or adding new subspecialist training in our country, we were able to exact systematic change by training non-specialist personnel to work together to identify and complete PC tasks in the PICU. Our programs have been massively successful in improving the quality and accessibility of PCC and PPC care to pediatric patients throughout Ecuador and further support the hypothesis IMOC is the best model to meet extensive PPC needs.

The extensive PC need in our context (20, 46, 49, 51, 55) and new data supporting the effectiveness of the IMOC (9) lead the principal investigator (Michelle Grunauer) to implement the IMOC in the PICU of the Hospital de los Valles (HDLV) when it was established in Quito, Ecuador in 2013. HDLV’s PICU was the first PICU in Ecuador to use a family-centered IMOC. She integrated the IMOC into the PICU because there is no known evidence that the external consultation strategy provides better patient care and because the IMOC is particularly well suited to under-resourced settings like ours, where there are no resources available to create an external PC service.

Michelle Grunauer incorporated the IMOC into this unit primarily through a certificate training program she launched in conjunction with physicians from the United States trained in PCCEM to elevate local medical professionals’ expertise in critical care and PC (54). “The Laude in PCCEM employs a family-centered approach by integrating pediatric critical care (PCC), mental health, and palliative medicine. It is innovative in that any critically ill (not merely dying) child benefits from family involvement [in an IMOC] so that family might understand the disease process and take part in treatment decisions” (54). In essence, participants learned how to provide care to critically ill patients according to the framework of the IMOC.

Various methods are effective in integrating health-care professionals into the IMOC. In the first program launched in HDLV, instructors used a combination of pre-readings, classroom instruction, bedside didactics, and simulation drills to teach professionals about the IMOC (54). These activities integrated PC and family-centered care techniques into PCCEM by focusing on case-based learning, interdisciplinary/interprofessional teamwork, individual confidence, and skill competency (54). The activities themselves emphasized techniques and tasks key to the IMOC, including communication, symptom management, planning, ethical dilemmas, and other topics. Integrating these themes into the standardly available Advanced Pediatric Life Support curriculum is also an effective introduction to the IMOC (54).

A number of indicators pointed to the IMOC’s success in HDLV’s PICU. Improved holistic care provided through the IMOC seems to have impacted the satisfaction of patients and families that are admitted; this PICU has not had a single medical lawsuit during its 5 years of existence. Staff appear to provide better care and patients seem to have better symptom management, greater familial involvement, and improved overall outcomes since the implementation of the IMOC (54). In addition, the unit’s mortality rate decreased from 10 to 4.8% after the staff were fully trained in the IMOC [Ecuador’s average PICU mortality rate is 15% (45)]. These unit-wide improvements suggest that the IMOC may better meet the extensive special care needs of HDLV’s PICU patients. Further studies are needed to determine the effectiveness of the IMOC in other settings, but this may be a better care model for other settings as well.

## Conclusion

This article has identified reasons why the IMOC is well designed to meet the extensive PPC needs in PICUs across different contexts, especially in under-resourced settings like Ecuador. Currently, the direct integration of PC into the PICU is met with resistance because of prevalent myths and misconceptions about PC, discipline-related bias, and ignorance of evidence-based recommendations related to the application of PC. However, evidence (4, 5, 31) indicates that virtually all patients admitted to the PICU have some level of PC need; thus, PICU models must make PC highly accessible to all patients who may develop special care needs throughout their stay in the unit. Training difficulties, lack of specialists, consultation barriers, and a number of other factors appear to impede the consultative model from meeting this goal both in industrialized contexts (6) and in the global south. Considering the propriety of the IMOC to meet extensive PPC needs across different settings, the authors recommend further research related to the establishment of the IMOC in other centers, the effects of this model on quality of care, and comparisons of the IMOC with the consultative model. Employing the IMOC in PICUs around the world could represent an important paradigm shift within pediatric intensive medicine which could elevate care to a superior level, especially in under-resourced contexts.

## Author Contributions

MG contributed to the following in the elaboration of this investigation:

– Substantial contributions to the conception or design of the work and the acquisition, analysis, or interpretation of data for the work.
– Critical revision for important intellectual content.
– Final approval of the version to be published.
– Agreement to be accountable for all aspects of the work in ensuring that questions related to the accuracy or integrity of any part of the work are appropriately investigated and resolved.

CM contributed to the following in the elaboration of this investigation:

– Substantial contributions to the conception or design of the work and the acquisition, analysis, or interpretation of data for the work.
– Drafting the work and revising it critically for important intellectual content.
– Final approval of the version to be published.
– Agreement to be accountable for all aspects of the work in ensuring that questions related to the accuracy or integrity of any part of the work are appropriately investigated and resolved.

## Conflict of Interest Statement

The authors declare that the research was conducted in the absence of any commercial or financial relationships that could be construed as a potential conflict of interest.

## Abbreviations

ICU, intensive care unit; PICU, pediatric intensive care unit; PC, palliative care; PPC, pediatric palliative care; IMOC, integrated model of care; DNR, do not resuscitate; WHO, World Health Organization; LMICs, low-middle income countries; HDLV, Hospital de los Valles; PCCEM, Pediatric Critical Care and Emergency Medicine; PaPaS, Pediatric Palliative Screening Scale.
